# Practical experience with initial quality assurance of a high dose rate brachytherapy grid‐based Boltzmann solver algorithm

**DOI:** 10.1002/acm2.14392

**Published:** 2024-05-14

**Authors:** Jessica M. Fagerstrom

**Affiliations:** ^1^ Department of Radiation Oncology University of Washington Seattle Washington USA; ^2^ Department of Radiation Oncology Kaiser Permanente Seattle Washington USA

**Keywords:** brachytherapy, commissioning, dose calculation, HDR, MBDCA, quality assurance, treatment planning

## Abstract

**Purpose:**

The purpose of this study was to validate the use of a model‐based dose calculation algorithm (MBDCA), Acuros BV, for high dose rate brachytherapy treatment planning for a community‐based hospital with a Bravos afterloader. Based on published AAPM recommendations, this work details a practical approach for community‐based clinics to complete initial validation of Acuros BV, in order to add a MBDCA to a TG‐43 based brachytherapy treatment planning program.

**Methods:**

Source dimensions and materials used in Acuros BV and TG‐43 source models were compared to the physical source. TG‐186 testing was completed with standardized test cases externally calculated with Monte Carlo compared to locally calculated with Acuros BV. Point doses calculated using TG‐43 were compared to those calculated with Acuros BV in water at various dose grid settings. Secondary dose check software was used to evaluate dose distributions resembling clinical patient plans, both in water and on CT datasets representative of patient anatomy.

**Results:**

The major source of discrepancy of source models was the length of modeled steel cable. TG‐186 testing showed that the largest differences between Monte Carlo and Acuros BV dose distributions were located along the source axis for cases calculated in water, as well as located in regions of high dose gradients and within the applicator for the case calculated with a generic shielded applicator. An audit of point doses calculated with both TG‐43 and Acuros BV in water found that dose grid settings significantly affected agreement. Secondary dose check software indicated that Acuros BV functioned satisfactorily, and a 5% threshold was adopted for secondary dose checks on gynecologic plans.

**Conclusion:**

This validation process indicated that Acuros BV met expected standards and affirmed its suitability for integration into this clinical practice's brachytherapy treatment planning.

## 1 INTRODUCTION

A community‐based hospital aimed to validate the use of a model‐based dose calculation algorithm (MBDCA) for high dose rate (HDR) brachytherapy dose planning for use with a Bravos afterloader (Varian Medical Systems, Inc., Palo Alto, CA). The Acuros BV algorithm is a Grid‐Based Boltzmann Solver (GBBS) and is available as a licensable module within the Brachytherapy Planning BrachyVision treatment planning system (Varian). Unlike the water‐based brachytherapy dose calculation formalism included in Task Group 43 and its updates,^1^, ^2^, ^3^ MBDCAs consider inhomogeneities in the dose deposition volume. The TG‐43 approximation does not consider finite patient dimensions or inhomogeneities present in patient anatomy or applicators, which can impact the resulting dose distribution. MBDCAs offer the ability to take these aspects into account.^4^

The American Association of Physicists in Medicine (AAPM) has previously published guidance on MBDCA adoption for brachytherapy, including Task Group 186^4^ and the AAPM Working Group on Model‐Based Dose Calculation Algorithms in Brachytherapy (WGDCAB) Report 372.^5^ The AAPM Medical Physics Practice Guideline (MPPG) 13.A on HDR brachytherapy does not include direction on MBDCAs, deferring to TG‐186. Note that the MPPG states: “Due to possible dosimetric implications on the treatment prescription, MBDCAs should not be used clinically without rigorous validation and substantial brachytherapy experience.”^6^ According to TG‐186, two levels of commissioning are required before the clinical implementation of MBDCAs for brachytherapy planning: reproducing TG‐43 dose parameters and testing advanced capabilities of MBDCAs.

Several quality dosimetric studies have been completed comparing TG‐43 and Acuros BV dose distributions in various use cases, including comparisons of D_2cm3_ values, doses at depth, mean volume doses, and Points A and B.^7^, ^8^, ^9^, ^10^ Reviewing these metrics was considered out of scope for this study. Other work has performed numerical and experimental evaluations of the algorithm.^11^, ^12^ The current work is not meant to add to this body of literature, but rather to describe the practical methods of validating the use of Acuros BV for use in a clinical setting with a Varian Bravos afterloader. The steps detailed within this work were completed following the conclusion of the acceptance testing procedure included in the vendor's guidelines for installation, and prior to clinical use.

In contrast to comprehensive evaluations undertaken after the initial release of an MBDCA, which often involve resource‐intensive endeavors like full independent Monte Carlo simulations with three‐dimensional gamma analyses for comparison of reference dose distributions to locally calculated dose distributions, this study represents a streamlined and practical approach tailored for small clinics seeking to integrate Acuros BV into their TG‐43 based brachytherapy service. By focusing on the practical aspects of validation specific to clinical implementation, this work provides an example for similar institutions aiming to review Acuros BV without the need for extensive resources or expertise in advanced computational methods. This work may help provide guidance for efficiently validating the utility of Acuros BV within the constraints of routine clinical practice, offering perspective for community‐based hospitals navigating the addition of an MBDCA to their brachytherapy planning tools. These methods follow steps outlined in TG‐372,^5^ and this work aims to provide a single‐institution, real‐world example of how to implement these recommendations in a clinical setting.

## 2 METHODS

### 2.1 Review of source dimensions and materials

Source models used by Acuros BV and the secondary dose verification software were compared with dimensions and materials of the physical source from the source manufacturer (Alpha‐Omega Services, Inc., Bellflower, CA). The source used in the Bravos system is the same source as the GammaMed HDR Plus ^192^Ir model (Varian).^13^ The source consists of an active cylindrical core surrounded by AISI 316L stainless steel. An AISI 316L capsule cover is welded on the distal side, and the source capsule is welded to a braided AISI 304 stainless steel cable on the proximal side. Note this model differs from the GammaMed 12i HDR and GammaMed Plus PDR sources models.

### 2.2 TG‐186 testing

Recommended calculation‐based testing discussed in TG‐186 was conducted. MD Anderson's Imaging and Radiation Oncology Core Houston Quality Assurance Center (IROC Houston) has produced a user's guide for Acuros BV algorithm testing,^14^ working with data for test cases prepared by the AAPM's WGDCAB. This working group produced test case dose distributions that were calculated using Monte Carlo N‐Particle v.6 (MCNP6) transport code and the WGDCAB ^192^Ir source model, which was designed specifically for MBDCA commissioning and differs in composition and dimensions from the physical Bravos source. Three of these test cases use no applicator, and one uses a virtual shielded cylinder applicator, which was also designed for MBDCA commissioning and does not correspond to a commercially available applicator. IROC Houston hosts a repository with the MCNP6 test case dose distributions, available for public use. Varian has included the WGDCAB source and virtual shielded cylinder applicator in Acuros BV, versions 13 and higher.

The following tasks were completed, following the IROC Houston's user's guide:

1. Test cases were downloaded from the IROC Houston repository.
2. Reference MCNP6 dose distributions were downloaded.
3. Cases were imported into BrachyVision (v. 15.511).
4. Doses were calculated locally using the Acuros BV algorithm.
5. Reference and locally calculated results were compared.

All TG‐186 tests were completed using a source, source model, and treatment unit that were generated within the treatment planning system solely for comparing MCNP6‐generated dose distributions with locally calculated GBBS dose distributions. All test cases used a voxelized model of a (20.1 × 20.1 × 20.1) cm^3^ water phantom inside either a larger water phantom (Case 1) or larger air phantom (Cases 2−4) with dimensions (50.1 × 50.1 × 50.1) cm^3^. Both the inner and outer cubes for all cases had their centers at position (0,0,0), with dimensions chosen such that they accommodated 511 × 511 × 511 cubic voxels at 1 mm^3^, as noted by the WGDCAB. As discussed previously, for Cases 1−3, no applicator was modeled. For Case 4, the WGDCAB tungsten‐shielded cylinder was used.^15^ This generic applicator has a tungsten‐alloy internal shield, a total length of 14.0 cm, and a radius of 1.8 cm. Table 1, summarizing the test case modeling geometries, is reproduced from the IROC Houston's user guide.

**TABLE 1:** Test case geometries for Acuros BV algorithm testing. Reproduced from IROC Houston's user guide. The source direction for this work corresponded with Varian's internal positive y‐axis (not positive z‐axis as detailed in the IROC Houston user's guide).

Test case	Inner cube material “Cube”	Outer cube material “BgBOX”	^192^Ir source center location	Applicator
1	H_2_O	H_2_O	(0,0,0) cm	None
2	H_2_O	Air	(0,0,0) cm	None
3	H_2_O	Air	(7,0,0) cm	None
4	H_2_O	Air	(0,0,0) cm	WG shielded cylinder

In BrachyVision, each plan was copied and then pasted into a new LOCAL_USER plan, and the date of the plan was changed to a date after the creation of the new brachytherapy model. Under the applicator, the afterloader was changed to “Bravos” and then back to “TG‐186” per Varian recommendations. Plan geometric parameters were verified with the IROC Houston's user guide. A TG‐43 calculation was run locally, which overrode the original dose, and then a local Acuros BV calculation was completed using a calculation medium set to the CT values, with the reporting medium set to water.

For all cases, the dose grid was set with an isotropic resolution of 0.1000 cm, with 201 pixels in all directions, centered on (0,0,0) to encompass the inner cube. All plans at this institution are calculated clinically using a nominal 40700 U source strength. This corresponds to a 10 Ci source using historical units, with a conversion coefficient for air kerma strength to Ci of 4.070 × 10^−3^ Gy m^2^/h/Ci from the vendor‐provided source certificate. This conversion coefficient matches the value included in the IROC Houston user's guide. Users should note that this conversion coefficient differs from the generic coefficient reported through Accredited Dosimetry Calibration Laboratories (ADCLs), which suggest using a value of 4.034 × 10^−3^ Gy m^2^/h/Ci. This ADCL conversion coefficient is included in the ADCL documentation for reference only, noting that AAPM TG‐43 replaced all historical source strength units with units of air kerma strength.^16^ Because the 4.034 × 10^−3^ Gy m^2^/h/Ci coefficient value is a generic coefficient from the ADCL and not a recommended factor for treatment planning, and because the 4.070 × 10^−3^ Gy m^2^/h/Ci coefficient value matches the IROC Houston guide as well as the source certificate, the source strength 40700 U was used for this work for consistency. Using the nominal source strength, nominal dwell times for a single dwell position were set to 10 s for Cases 1−3, and 50 s for Case 4. The resulting locally calculated dose distributions were compared with the MCNP6 distributions within the BrachyVision software.

### 2.3 Treatment planning system point dose audit

After comparing the locally calculated test cases with the reference MCNP6 calculations, TG‐43 data was used to review point doses of Acuros BV calculations within BrachyVision. Ballester et al.^17^ tabulated values for TG‐43 formalism for a GammaMed Plus ^192^Ir HDR source, calculated using GEANT Monte Carlo. Taylor and Rogers^18^ later independently determined TG‐43 data values with EGSnrc Monte Carlo, using the same source model as Ballester et al. A consensus dataset combining both studies was put forth by the High Energy Brachytherapy Source Dosimetry (HEBD) Working Group via a joint report through AAPM and ESTRO.^19^ For this work, HEBD TG‐43 calculated point doses were compared to Acuros BV calculations, generated using various dose grid settings.

Within a new test patient in BrachyVision, a single dwell position was placed at the center of a homogeneous (25 × 25 × 25) cm^3^ water phantom, with a nominal dwell time of 3600.0 s (1 h) and a nominal source strength of 40700 U. The plan was calculated using the Acuros BV algorithm as described above, using the source “BRAVOS,” which will be used for patient treatment (not the TG‐186 source used only for TG‐186 testing). The calculation medium was set to CT values, and the reporting medium was set to water (the medium is the same as water for this phantom). The dose grid was set at resolutions of (0.25 × 0.25 × 0.25) cm^3^, (0.1 × 0.1 × 0.125) cm^3^, (0.0625 × 0.0625 × 0.0625) cm^3^, and (0.05 × 0.05 × 0.05) cm^3^, and it was set to encompass the entire water phantom for testing.

### 2.4 Secondary dose check software validation

The institution's brachytherapy secondary dose verification software, RadCalc (LifeLine Software, Austin, TX), was reviewed for use with Acuros BV and as an additional tool to validate the GBBS. The RadCalc brachytherapy module uses the TG‐43 algorithm, and was configured to use TG‐43 data derived from Ballester et al.^17^ Multiple Acuros BV plans were exported to RadCalc, with RadCalc values for nominal planned and treatment source strength set to 40700 U. First, point dose was calculated by RadCalc and compared to that calculated by Acuros BV in water for a templated interstitial gynecologic plan. This template was created from previous patient data. It contained one calculation point as the recto‐vaginal point,^20^ and included 13 applicators in the geometric configuration of a single tandem and 12 interstitial needles for a cervix implant with 71 dwell positions. The plan did not contain virtual solid applicator models. This templated plan was imported into a water phantom for Acuros BV dose calculation, then exported to RadCalc.

Following the analysis of the templated plan with the homogeneous water phantom, a total of 15 anonymized brachytherapy patient plans (for patients who had completed treatment) were recalculated with Acuros BV. The original structure sets were copied, then the physical material table was assigned as AcurosXB‐13.5. The dose grids were set to (0.0625 × 0.0625 × 0.0625) cm^3^ surrounding the region of interest. The calculation medium was set as the CT values, and the reporting medium was set as the medium. The plans were all for gynecologic patients with low‐Z applicators: five single‐channel, unshielded vaginal cylinder plans, and ten hybrid intracavitary/interstitial cervix plans. The cylinder applicators for this clinical site are composed of plastics polyetheretherketone (PEEK) and polyphenylsulfone (PPSU), along with some titanium elements located outside of the field of view. The cervix implants use disposable needles composed of plastic and intrauterine tandems composed of a proprietary polymer material. Standard practice for this clinical site for intracavitary single‐channel, unshielded vaginal cylinder treatments is to include one dose calculation point lateral to the applicator at a depth of 0.5 cm from the applicator surface, and one calculation point along the cylinder/source axis, also at a depth of 0.5 cm from the distal end of the applicator. These same dose points were used for Acuros BV‐RadCalc comparisons. Standard practice for this clinical site for interstitial cervical treatments is to include one calculation point for the secondary check software (the recto‐vaginal point). The majority of these patients are implanted twice, 1 week apart, and have at least two different plans generated, each on a different CT dataset. These calculation points were used for Acuros BV‐RadCalc comparisons.

## 3 RESULTS

### 3.1 Review of source dimensions and materials

Dimensions and compositions from the manufacturer were compared to those used within Acuros BV, the HEBD consensus dataset, and the secondary check software source models. Varian Medical System's BrachyVision Algorithms Reference Guide details the geometry of the GammaMed Plus HDR source used in their model.^21^ This geometry was compared to the schematic included in the HEBD report. The HEBD and BrachyVision sources for the GammaMed HDR Plus match each other and information from the manufacturer, except for the woven stainless steel cable. Ballester et al.^17^ and the HEBD Monte Carlo data both model the woven stainless steel cable as a 6 cm long, stainless steel cylinder. In Acuros BV, the steel cable is only modeled 0.2 cm back from the source capsule, because this is the maximum length at which the manufacturer determined that significant bending would not likely occur. Based on these discrepancies, it is expected that dose points found using water‐based brachytherapy dose calculation formalism compared to Acuros BV will vary along the source axis, most significantly within the cable volume, as previously noted in the literature.^22^ This is especially noticeable in the section detailing calculation point dose auditing.

### 3.2 TG‐186 testing

**FIGURE 1:**
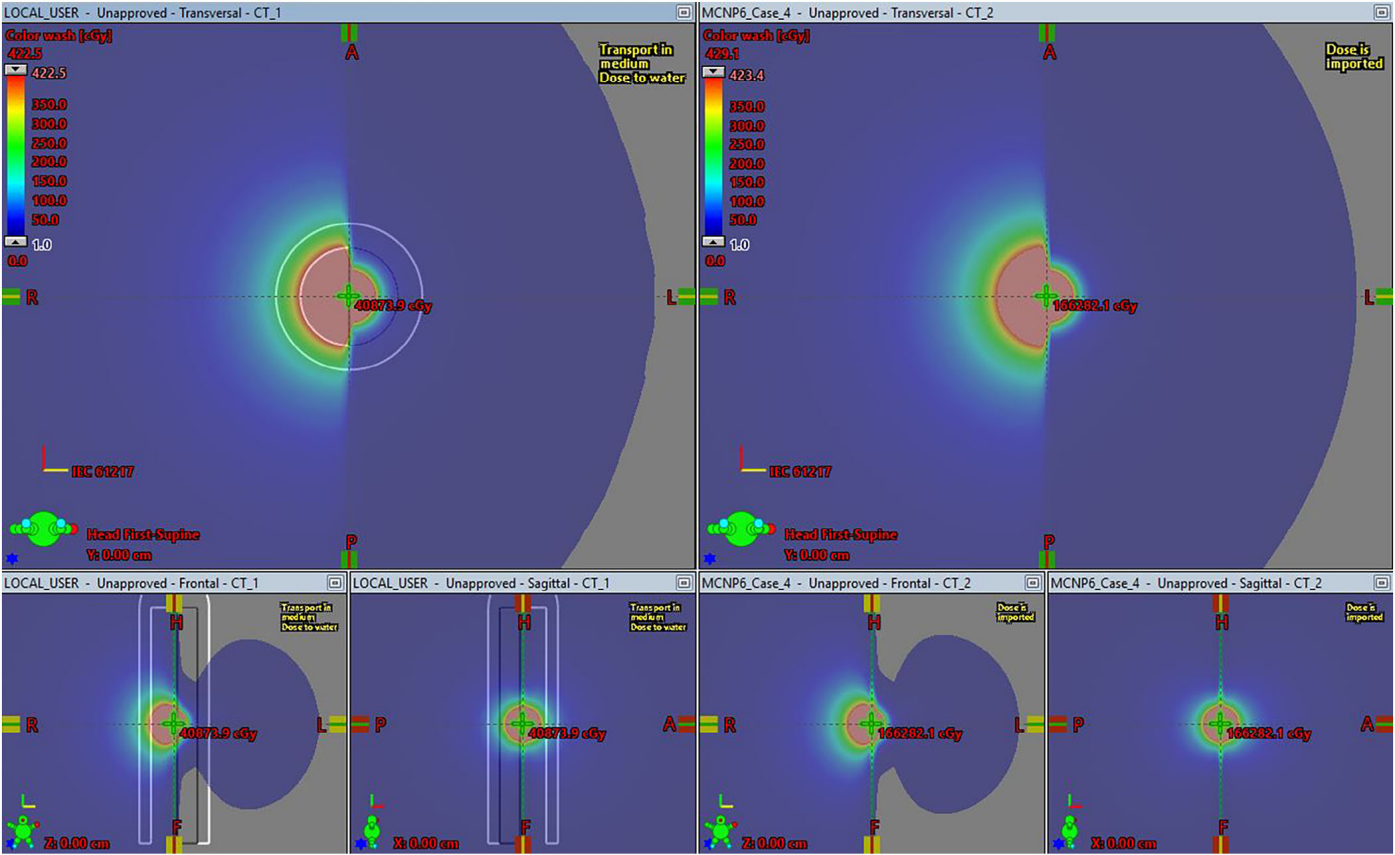
TG‐186 Case 4, with the locally calculated Acuros BV distribution on the left, and the MCNP6 distributions on the right. Dose color wash range is set equal for both cases.

**FIGURE 2:**
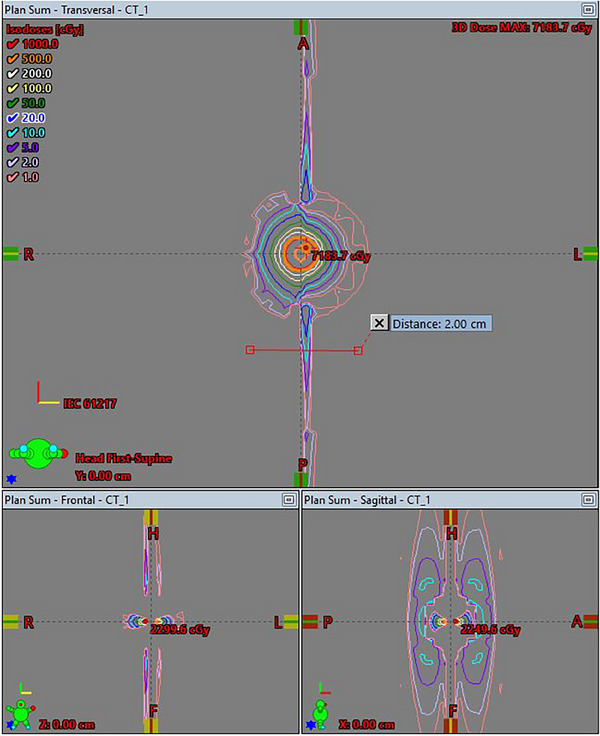
Two‐dimensional planar views of TG‐186 Case 4 dose difference plan sum, with the MCNP6‐generated imported dose subtracted from the locally calculated Acuros BV dose. A distance of 2.0 cm is delineated in the axial plane for reference.

**FIGURE 3:**
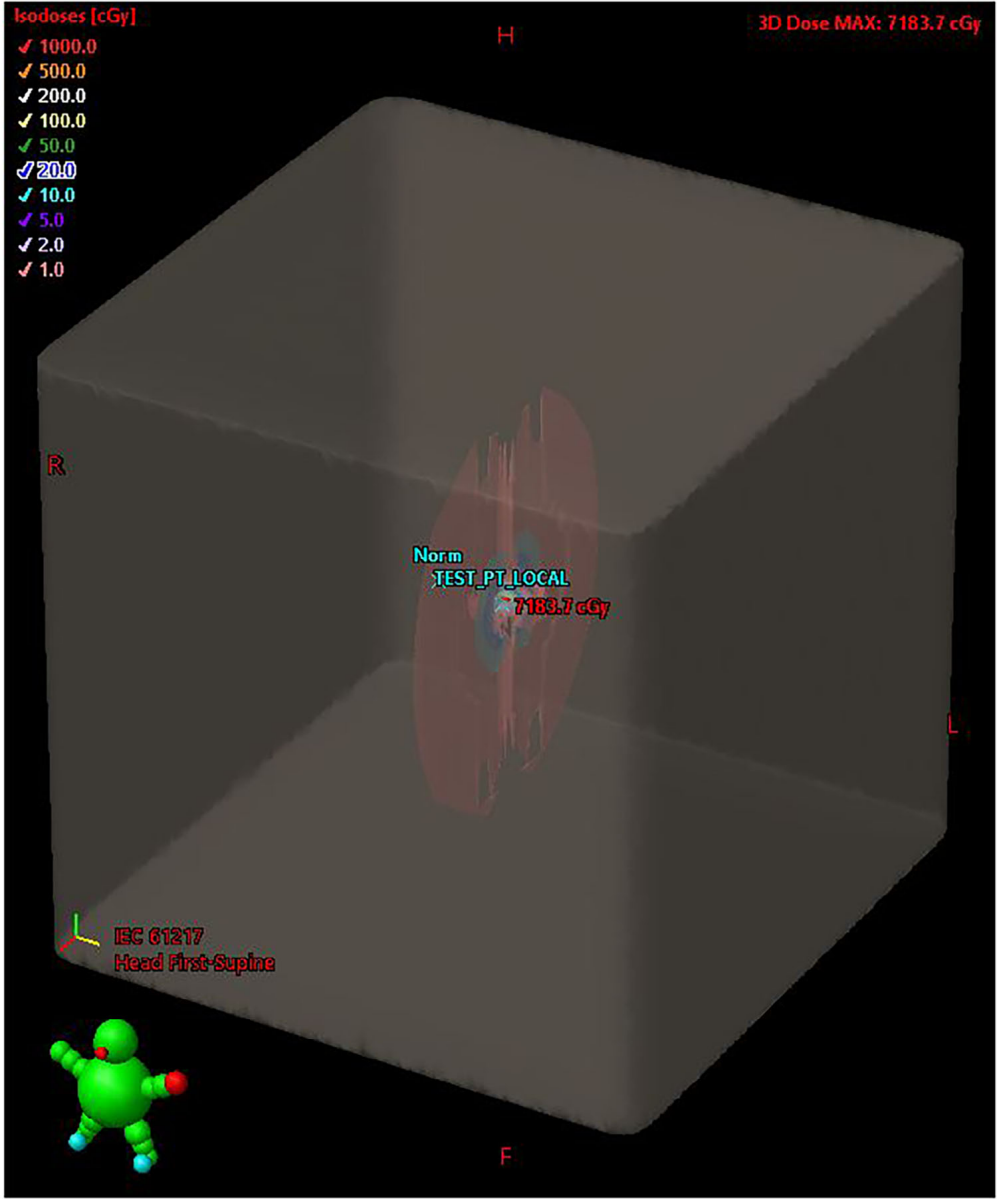
Three‐dimensional view of TG‐186 Case 4 dose difference plan sum, with the MCNP6‐generated imported dose subtracted from the locally calculated Acuros BV dose.

For each of the test cases included in TG‐186, dose ratios were used to compare dose at selected points of the locally calculated Acuros BV distributions to those of the imported MCNP6 distributions. These points are included in Tables 2, 3, 4, 5. The distributions calculated locally with Acuros BV were compared to the MCNP6 distributions in Eclipse's Plan Evaluation platform (Varian). Plan sums were generated subtracting the MCNP6‐generated imported dose from the locally calculated Acuros BV dose. Selected views of this comparison are included in Figures 1, 2, and 3 for Case 4 as an example.

**TABLE 2:** Dose differences for selected calculation points, comparing locally calculated Acuros BV to MCNP6 for TG‐186 Case 1, a single source dwell in the center of an inner water cube situated within an outer water cube. The y‐axis is along the source axis. Points B and E are located along the source axis and Points J–L represent low dose regions.

	Point position					
Point	x (cm)	y (cm)	z (cm)	Locally calculated Acuros BV dose (cGy)	MCNP6 dose (cGy)	Percent difference (%)
A	1.0	0.0	0.0	125.6	125.6	0.0%
B	0.0	1.0	0.0	82.6	82.7	−0.1%
C	0.0	0.0	1.0	125.5	125.6	−0.1%
D	−1.0	0.0	0.0	125.5	125.6	−0.1%
E	0.0	−1.0	0.0	76.4	77.0	−0.8%
F	0.0	0.0	−1.0	125.6	125.6	0.0%
G	0.0	−2.0	2.0	15.4	15.4	0.0%
H	−0.5	−0.5	−0.5	165.8	165.9	−0.1%
I	0.5	0.5	0.5	165.8	165.7	0.1%
J	8.0	0.0	0.0	1.9	1.9	0.0%
K	0.0	8.0	0.0	1.4	1.4	0.0%
L	0.0	0.0	−8.0	1.9	1.9	0.0%

**TABLE 3:** Dose differences for selected calculation points, comparing locally calculated Acuros BV to MCNP6 for TG‐186 Case 2, a single source dwell in the center of an inner water cube situated within an outer cube of air. The y‐axis is along the source axis. Points B and E are located along the source axis and Points J–L represent low dose regions.

	Point position					
Point	x (cm)	y (cm)	z (cm)	Locally calculated Acuros BV dose (cGy)	MCNP6 dose (cGy)	Percent difference (%)
A	1.0	0.0	0.0	125.9	125.5	0.3%
B	0.0	1.0	0.0	84.9	82.5	2.9%
C	0.0	0.0	1.0	125.0	125.4	−0.3%
D	−1.0	0.0	0.0	126.1	125.4	0.6%
E	0.0	−1.0	0.0	78.7	76.3	3.1%
F	0.0	0.0	−1.0	125.2	125.4	−0.2%
G	0.0	−2.0	2.0	15.3	15.2	0.7%
H	−0.5	−0.5	−0.5	165.4	165.7	−0.2%
I	0.5	0.5	0.5	165.3	165.6	−0.2%
J	8.0	0.0	0.0	1.8	1.8	0.0%
K	0.0	8.0	0.0	1.3	1.3	0.0%
L	0.0	0.0	−8.0	1.8	1.8	0.0%

**TABLE 4:** Dose differences for selected calculation points, comparing locally calculated Acuros BV to MCNP6 for TG‐186 Case 3, a single source dwell shifted 7 cm from the center of an inner water cube situated within an outer water cube. The y‐axis is along the source axis. Points A–L correspond to the same distances relative to the source dwell position as those used for Cases 1, 2, and 4. Points B and E are located along the source axis and Points J–L represent low dose regions.

	Point position					
Point	x (cm)	y (cm)	z (cm)	Locally calculated Acuros BV dose (cGy)	MCNP6 dose (cGy)	Percent difference (%)
A	8.0	0.0	0.0	125.3	125.2	0.1%
B	7.0	1.0	0.0	83.8	82.3	1.8%
C	7.0	0.0	1.0	125.1	125.2	−0.1%
D	6.0	0.0	0.0	125.6	125.3	0.2%
E	7.0	−1.0	0.0	77.5	76.1	1.8%
F	7.0	0.0	−1.0	125.1	125.3	−0.2%
G	7.0	−2.0	2.0	15.1	15.1	0.0%
H	6.5	−0.5	−0.5	165.2	165.5	−0.2%
I	7.5	0.5	0.5	165.0	165.5	−0.3%
J	1.0	0.0	0.0	3.4	3.4	0.0%
K	7.0	8.0	0.0	1.2	1.2	0.0%
L	7.0	0.0	−8.0	1.7	1.7	0.0%

**TABLE 5:** Dose differences for selected calculation points, comparing locally calculated Acuros BV to MCNP6 for TG‐186 Case 4, a single source dwell within the WG shielded cylinder in the center of an inner water cube situated within an outer cube of air. The y‐axis is along the source axis. Points B and E are located along the source axis and Points J–L represent low dose regions. Points A–F, H, and I fall within the applicator volume.

	Point position					
Point	x (cm)	y (cm)	z (cm)	Locally calculated Acuros BV dose (cGy)	MCNP6 dose (cGy)	Percent difference (%)
A	1.0	0.0	0.0	74.1	73.3	1.1%
B	0.0	1.0	0.0	378.9	480.1	−21.1%
C	0.0	0.0	1.0	316.0	298.3	5.9%
D	−1.0	0.0	0.0	623.4	621.3	0.3%
E	0.0	−1.0	0.0	349.2	435.8	−19.9%
F	0.0	0.0	−1.0	315.2	298.3	5.7%
G	0.0	−2.0	2.0	30.5	32.3	−5.6%
H	−0.5	−0.5	−0.5	799.2	809.0	−1.2%
I	0.5	0.5	0.5	137.7	142.1	−3.1%
J	8.0	0.0	0.0	0.9	0.9	0.0%
K	0.0	8.0	0.0	3.7	6.0	−38.3%
L	0.0	0.0	−8.0	3.7	4.6	−19.6%

For the dose ratio comparisons, in Cases 1−3, the largest dose differences appeared along the source axis, near the source (the center of the source is located 0.25 cm from the distal end of the source wire). For Case 4, large differences were also seen for point positions located in regions of high dose gradients or within the applicator, inside the shielding. An examination of the two‐ and three‐dimensional dose difference distributions reiterated the dose ratio comparison results across the four test cases. The dose difference distributions indicated minimal discrepancies, with notable exceptions in regions immediately adjacent to the radiation source. These localized differences were anticipated, as previously discussed. The dose difference maps were reviewed with the lead brachytherapy attending physician, who affirmed the clinical viability of the Acuros BV algorithm for brachytherapy treatment planning. This qualitative decision was informed by the clinician's experience, who prioritized practical considerations in real‐world scenarios over precise dose measurements for points in very close proximity to the radiation source, where the observed dose disparities in this context were expected. Readers are encouraged to consult with their respective clinicians to ascertain clinically acceptable dose variations in their specific circumstances. Based on this work, the two‐ and three‐dimensional dose difference distributions were considered clinically acceptable in all four cases.

### 3.3 Treatment planning system point dose audit

The point dose audit demonstrated noticeable variations in calculated dose to reference points based on dose grid resolution, as discussed in the BrachyVision Algorithms Reference Guide and TG‐186. These calculated doses were compared to those reported by the HEBD Working Group, and results of the spot checks are included in Tables 6 and 7. Disagreement between the HEBD and Acuros BV values was high within the volume of the braided steel cable. The steel cable is not modeled in Acuros BV beyond 0.2 cm, but it is included in the HEBD data.

**TABLE 6:** Acuros BV point doses compared to point doses included in HEBD's Table XVII. The y‐axis is along the source axis. The steel cable is not modeled in Acuros BV beyond 0.2 cm, but it is included in the HEBD data. Points Q and AA (*) are points located within the cable volume. Point FF (**) represents the maximum distance from the source included in HEBD Table XVII.

	Point position			TPS‐calculated values (cGy/hr/U)					
Point	x (cm)	y (cm)	z (cm)	0.25 × 0.25 × 0.25 cm^3^ dose grid	0.1 × 0.1 × 0.1 cm^3^ dose grid	0.0625 × 0.0625 × 0.0625 cm^3^ dose grid	0.05 × 0.05 × 0.05 cm^3^ dose grid	HEBD values (cGy/hr/U)	Distance from source center (cm)
A	0.25	0.00	0.00	15.1329	15.0374	15.6982	15.5938	15.70	0.25
B	−0.25	0.00	0.00	15.1329	15.0374	15.6982	15.5938	15.70	0.25
C	0.00	0.00	0.25	15.1329	15.0374	15.6982	15.5938	15.70	0.25
D	0.00	0.00	−0.25	15.1329	15.0374	15.6982	15.5938	15.70	0.25
E	0.50	0.00	0.00	4.6311	4.3451	4.3097	4.2994	4.32	0.50
F	−0.50	0.00	0.00	4.6311	4.3451	4.3097	4.2994	4.32	0.50
G	0.00	0.00	0.50	4.6311	4.3451	4.3097	4.2994	4.32	0.50
H	0.00	0.00	−0.50	4.6311	4.3451	4.3097	4.2994	4.32	0.50
I	0.25	0.50	0.00	3.2753	3.4101	3.3983	3.4027	3.47	0.56
J	0.50	0.50	0.00	2.1907	2.1818	2.1791	2.1788	2.21	0.71
K	0.00	0.50	−0.50	2.1907	2.1818	2.1810	2.1788	2.21	0.71
L	0.75	0.00	0.00	2.0372	1.9425	1.9580	1.9557	1.966	0.75
M	−0.75	0.00	0.00	2.0372	1.9425	1.9580	1.9557	1.966	0.75
N	0.00	0.00	0.75	2.0372	1.9425	1.9580	1.9557	1.966	0.75
O	0.00	0.00	−0.75	2.0372	1.9425	1.9580	1.9557	1.966	0.75
P	0.00	1.00	0.00	0.8366	0.7660	0.7449	0.7405	0.707	1.00
Q (*)	0.00	−1.00	0.00	0.8125	0.7109	0.6855	0.6802	0.505	1.00
R	1.00	0.00	0.00	1.1383	1.1138	1.1115	1.1107	1.117	1.00
S	−1.00	0.00	0.00	1.1383	1.1138	1.1115	1.1107	1.117	1.00
T	0.00	0.00	1.00	1.1383	1.1138	1.1115	1.1107	1.117	1.00
V	0.00	0.00	−1.00	1.1383	1.1138	1.1115	1.1107	1.117	1.00
W	0.00	1.50	0.00	0.3529	0.3330	0.3292	0.3278	0.323	1.50
X	0.00	2.00	0.00	0.1918	0.1811	0.1789	0.1782	0.1798	2.00
Y	0.00	3.00	3.00	0.0601	0.0601	0.0601	0.0601	0.0607	4.24
Z	0.00	5.00	0.00	0.0301	0.0312	0.0312	0.0312	0.0317	5.00
AA (*)	0.00	−5.00	0.00	0.0301	0.0297	0.0296	0.0296	0.0236	5.00
BB	5.00	0.00	0.00	0.0447	0.0447	0.0447	0.0447	0.0451	5.00
CC	−5.00	0.00	0.00	0.0447	0.0447	0.0447	0.0447	0.0451	5.00
DD	0.00	0.00	5.00	0.0447	0.0447	0.0447	0.0447	0.0451	5.00
EE	0.00	0.00	−5.00	0.0447	0.0447	0.0447	0.0447	0.0451	5.00
FF (**)	0.00	−7.00	7.00	0.0098	0.0098	0.0098	0.0098	0.01046	9.90

**TABLE 7:** Acuros BV point doses compared to point doses included in HEBD's Table XVII. The y‐axis is along the source axis. The steel cable is not modeled in Acuros BV beyond 0.2 cm, but it is included in the HEBD data. Points Q and AA (*) are points located within the cable volume. Point FF (**) represents the maximum distance from the source included in HEBD Table XVII.

	Point position			HEBD values percent difference from TPS‐calculated values			
Point	x (cm)	y (cm)	z (cm)	0.25 × 0.25 × 0.25 cm^3^ dose grid	0.1 × 0.1 × 0.1 cm^3^ dose grid	0.0625 × 0.0625 × 0.0625 cm^3^ dose grid	0.05 × 0.05 × 0.05 cm^3^ dose grid
A	0.25	0.00	0.00	−3.6%	−4.2%	0.0%	−0.7%
B	−0.25	0.00	0.00	−3.6%	−4.2%	0.0%	−0.7%
C	0.00	0.00	0.25	−3.6%	−4.2%	0.0%	−0.7%
D	0.00	0.00	−0.25	−3.6%	−4.2%	0.0%	−0.7%
E	0.50	0.00	0.00	7.2%	0.6%	−0.2%	−0.5%
F	−0.50	0.00	0.00	7.2%	0.6%	−0.2%	−0.5%
G	0.00	0.00	0.50	7.2%	0.6%	−0.2%	−0.5%
H	0.00	0.00	−0.50	7.2%	0.6%	−0.2%	−0.5%
I	0.25	0.50	0.00	−5.6%	−1.7%	−2.1%	−1.9%
J	0.50	0.50	0.00	−0.9%	−1.3%	−1.4%	−1.4%
K	0.00	0.50	−0.50	−0.9%	−1.3%	−1.3%	−1.4%
L	0.75	0.00	0.00	3.6%	−1.2%	−0.4%	−0.5%
M	−0.75	0.00	0.00	3.6%	−1.2%	−0.4%	−0.5%
N	0.00	0.00	0.75	3.6%	−1.2%	−0.4%	−0.5%
O	0.00	0.00	−0.75	3.6%	−1.2%	−0.4%	−0.5%
P	0.00	1.00	0.00	18.3%	8.3%	5.4%	4.7%
Q (*)	0.00	−1.00	0.00	60.9%	40.8%	35.7%	34.7%
R	1.00	0.00	0.00	1.9%	−0.3%	−0.5%	−0.6%
S	−1.00	0.00	0.00	1.9%	−0.3%	−0.5%	−0.6%
T	0.00	0.00	1.00	1.9%	−0.3%	−0.5%	−0.6%
V	0.00	0.00	−1.00	1.9%	−0.3%	−0.5%	−0.6%
W	0.00	1.50	0.00	9.3%	3.1%	1.9%	1.5%
X	0.00	2.00	0.00	6.7%	0.7%	−0.5%	−0.9%
Y	0.00	3.00	3.00	−1.0%	−1.0%	−1.0%	−1.0%
Z	0.00	5.00	0.00	−5.0%	−1.6%	−1.6%	−1.6%
AA (*)	0.00	−5.00	0.00	27.5%	25.8%	25.4%	25.4%
BB	5.00	0.00	0.00	−0.9%	−0.9%	−0.9%	−0.9%
CC	−5.00	0.00	0.00	−0.9%	−0.9%	−0.9%	−0.9%
DD	0.00	0.00	5.00	−0.9%	−0.9%	−0.9%	−0.9%
EE	0.00	0.00	−5.00	−0.9%	−0.9%	−0.9%	−0.9%
FF (**)	0.00	−7.00	7.00	−6.3%	−6.3%	−6.3%	−6.3%

### 3.4 Secondary dose check software validation

For the Acuros BV‐RadCalc comparison of the templated cervix plan in the homogeneous water phantom, the Acuros BV calculated recto‐vaginal point was 9.5042 Gy, and RadCalc TG‐43‐based point was 9.8989 Gy (−1.0% difference). For the five anonymized vaginal cylinder intracavitary plans and the ten anonymized hybrid interstitial/intracavitary plans calculated on CT datasets with Acuros BV and using TG‐43 in RadCalc, point dose comparisons are included in Tables 8 and 9. Considering these results, it was noted that Point 2 for Patient 4 was shifted approximately 0.2 cm laterally from the cylinder axis, so was not geometrically located on the axis as was the case for the other plans’ Point 2. For all other comparisons, Point 2 was correctly placed along the source and applicator axis. Points along the source and applicator axis exhibit larger differences between MBDCA and TG‐43 calculations, as expected. The unexpectedly good agreement for Point 2, Patient 4 signaled its misplacement (it was not placed directly on the source axis), as well as illustrated that points near, but slightly displaced, from the source axis, demonstrate excellent agreement.

**TABLE 8:** Comparison of Acuros BV‐calculated and RadCalc‐calculated point doses for completed single‐channel vaginal cylinder plans. Point 1 is located 0.5 cm lateral from the cylinder surface, and Point 2 is located 0.5 cm from the tip of the cylinder, along the source axis.

Patient	Acuros BV dose Point 1 (cGy)	RadCalc dose Point 1 (cGy)	Percent difference (%)	Acuros BV dose Point 2 (cGy)	RadCalc dose Point 2 (cGy)	Percent difference (%)
1	93.67	96.45	3.0%	70.06	65.58	−6.4%
2	87.53	89.70	2.5%	75.18	71.06	−5.5%
3	93.46	95.59	2.3%	75.21	69.87	−7.1%
4	93.12	95.06	2.1%	81.40	81.82	0.5%
5	92.19	95.41	3.5%	76.08	69.83	−8.2%

**TABLE 9:** Comparison of Acuros BV‐calculated and RadCalc‐calculated point doses for completed interstitial cervical plans. All patients below had two implants and two separate plans, each with a distinct calculation point.

Patient	Acuros BV dose point implant 1 (cGy)	RadCalc dose point implant 1 (cGy)	Percent difference (%)	Acuros BV dose point implant 2 (cGy)	RadCalc dose point implant 2 (cGy)	Percent difference (%)
6	537.09	535.43	−0.3%	546.38	545.04	−0.2%
7	567.02	581.52	2.6%	549.86	560.23	1.9%
8	512.20	526.22	2.7%	534.89	549.18	2.7%
9	594.79	605.36	1.8%	321.83	326.49	1.4%
10	515.25	531.50	3.2%	545.50	562.17	3.1%

## 4 DISCUSSION

During this evaluation, some significant differences in doses calculated by Acuros BV and TG‐43 were evident. These were especially pronounced on the axis of the source, which has been reported by other authors (see TG‐186 for a summary^15^). Large differences in calculated dose were expected in the braided stainless steel cable region, based on differences between Acuros BV's model compared to Monte Carlo models used to determine TG‐43 parameters (0.2 cm length vs. 6 cm length). Differences along the source longitudinal axis, distal to the source wire (the opposite direction of the stainless steel cable) are explained by the anisotropy values determined by the TG‐43 algorithm within BrachyVision, as discussed by Papagiannis et al.^23^ TG‐186 recommends reporting both TG‐43‐ and MBDCA‐calculated brachytherapy dose distributions if MBDCAs are to be used clinically. Based on the review of secondary dose calculation data, it was decided to use a value of 5% absolute dose difference as a threshold for routine point‐based secondary check verifications in RadCalc. This assumes the calculation point is not located on the source axis—for vaginal cylinder plans, it was decided that a second point aside from the point placed at 0.5 cm from the cylinder distal end should be used. RadCalc was not expected to flag major dose discrepancies for the cases studied (pelvic brachytherapy in the absence of major inhomogeneities), but was reviewed for completeness. Secondary dose check results and thresholds should be revisited when adding additional applicators and treatment sites to clinical service.

Presently, the HDR service at this site exclusively involves the treatment of gynecologic cases using a suite of MRI‐compatible applicators and needles. The clinic was using the water‐based TG‐43 formalism to calculate dose for these plans at the time of this work, and now retrospectively reviews selected cases calculated with Acuros BV. While the utilization of MBDCAs with MRI‐compatible applicators for gynecologic treatments poses fewer challenges than many other clinical environments due to the absence of significant inhomogeneities, patient boundaries, and high atomic number materials, it is helpful to proactively assess and validate Acuros BV's performance. This evaluation can aid in rolling out future treatment sites, including those for which the differences between MBDCA and TG‐43 formalism calculations are expected to be larger. For these future cases, both Acuros BV and TG‐43 distributions will be reported. As the clinic considers extending its HDR service to include treatment sites that may involve significant inhomogeneities and/or high atomic number materials, cautious consideration will be given to the integration of the Acuros BV algorithm. While the current study has laid the foundation through the commissioning efforts detailed in this work, future implementation of new treatment sites will warrant careful scrutiny.

Some safety concerns were taken into account for the generation of the TG‐186 testing environment. The TG‐186 source model must not be used for patient calculations, so steps were taken to avoid this potential safety issue. A new radioactive source model was created with Source ID “TG‐186” and the Source Model “TG‐186(2015)”, and a new Treatment Unit was created called “TG‐186” with Varian's ARIA RT Administration. Prior to performing calculations for test cases using the TG‐186 option, the system displays warning messages. Following calculation, reports returned the following error messages: “ERROR: The radioactive source model used in the plan is not approved for treatment” and “ERROR: Operating status is not ‘Ready’ for the selected afterloader.” The operational status of the unit was set to “Virtual” and the source model status was set to “Commissioning” upon the completion of testing, in order to avoid using it with patient data. The treatment unit is no longer selectable when creating new plans.

There are some considerations in acquiring CT scans of patients when using Acuros BV. BrachyVision assumes the CT grid boundaries are non‐reentrant, meaning that photons can only exit CT grid boundaries, but cannot reenter them (so backscatter is not considered). Adequate CT volume must therefore be included in the region of the dose calculation. The results of the current work also informed CT slice thickness and dose grid resolution. Based on the calculations using varying dose grid settings, this institution opted to acquire images at 0.625 mm slice thickness, and to perform all final Acuros BV patient dose calculations using a (0.0625 × 0.0625 × 0.0625) cm^3^ dose grid. Such calculations are impractical for iterative dose optimization, so it was decided to use TG‐43 during optimization prior to final Acuros BV dose distribution calculation.

The analysis of the TG‐186 test cases primarily involved a basic examination of the differences between the MCNP‐calculated and the locally computed Acuros BV dose distributions, with the review of two‐dimensional and three‐dimensional dose maps and the determination of point dose ratios. A series of formal gamma analyses was not conducted based on the resources available at the institution. Formal gamma analyses comparing Acuros BV to TG‐186 test cases have previously been conducted and documented (Tien and Chen^24^). Instead, the primary objective of the TG‐186 test case work was to identify potentially significant issues within the MBDCA within the clinical environment in question. Substantial issues were not anticipated given the algorithm's duration of commercial availability; however, scrutiny of the MBDCA in the specific clinical context of the institution helps identify issues that may arise in practical applications.

Absorbed dose calculated with Acuros BV may be reported either to the local tissue at each voxel (denoted *D*_m,m_ in TG‐186), or to water (denoted *D*_w,m_ in TG‐186). These distributions are calculated the same, but then cavity theory is used to convert *D*_m,m_ to *D*_w,m_. The debate regarding *D*_m,m_ versus *D*_w,m_ is detailed in AAPM's TG‐105 in the external beam context, with no definitive recommendation given.^25^ In the context of brachytherapy, significant differences may be found between *D*_w,m_, *D*_m,m_, and doses derived from TG‐43 formalism (denoted *D*_w,w‐TG43_ in TG‐186). As an interim recommendation, TG‐186 recommends using *D*_m,m_ because it is a physical, well‐defined quantity and not a theoretical construct like *D*_w,m_. The task group authors recommend the area for further future research. With this in mind, this clinical site decided that when using Acuros BV, the calculation medium would be set as the CT values, and the reporting medium set as the medium.

## 5 CONCLUSION

The goal of this work was to provide a straightforward, practical example of the type of study used to validate an MBDCA, highlighting the importance of evaluating various components of a brachytherapy treatment planning algorithm. This work discusses the procedures followed at a community clinic to assess the performance of Acuros BV, focusing on the practical testing process of various aspects of the MBDCA. The TG‐186 quality assurance process involved comparing locally calculated dose distributions to MCNP6 distributions of established test cases. Treatment planning system point dose spot checks compared Acuros BV data to TG‐43 data, both directly from the High Energy Brachytherapy Source Dosimetry Working Group and by using secondary dose check software RadCalc. The initial quality assurance process of Acuros BV can provide valuable insight to clinical practices, empowering them to make informed decisions regarding the integration of MBDCAs into their own brachytherapy service.

## CONFLICT OF INTEREST STATEMENT

The author declares no conflicts of interest.

## References

[1] Nath R , Anderson LL , Luxton G , Weaver KA , Williamson JF , Meigooni AS . Dosimetry of interstitial brachytherapy sources: Recommendations of the AAPM Radiation Therapy Committee Task Group No. 43. Med Phys. 1995;22(2):209‐234. doi:10.1118/1.597458 7565352

[2] Rivard MJ , Coursey BM , DeWerd LA , et al. Update of AAPM Task Group No. 43 Report: A revised AAPM protocol for brachytherapy dose calculations. Med Phys. 2004;31(3):633‐674. doi:10.1118/1.1646040 15070264

[3] Rivard MJ , Butler WM , DeWerd LA , et al. Supplement to the 2004 update of the AAPM Task Group No. 43 Report. Med Phys. 2007;34(6Part1):2187‐2205. doi:10.1118/1.2736790 17654921

[4] Beaulieu L , Carlsson Tedgren A , Carrier JF , et al. Report of the Task Group 186 on model‐based dose calculation methods in brachytherapy beyond the TG‐43 formalism: current status and recommendations for clinical implementation. Med Phys. 2012;39(10):6208‐6236. doi:10.1118/1.4747264 23039658

[5] Beaulieu L , Ballester F , Granero D , et al. AAPM WGDCAB Report 372: A joint AAPM, ESTRO, ABG, and ABS report on commissioning of model‐based dose calculation algorithms in brachytherapy. Med Phys. 2023;50(8):e946‐e960. doi:10.1002/mp.16571 37427750

[6] Richardson SL , Buzurovic IM , Cohen GN , et al. AAPM Medical Physics Practice guideline 13.a: HDR brachytherapy, part A. J Appl Clin Med Phys. 2023;24(3):e13829. doi:10.1002/acm2.13829 36808798 PMC10018677

[7] Hira M , Podgorsak MB , Jaggernauth W , Malhotra HK . Measurement of dose perturbation around shielded ovoids in high‐dose‐rate brachytherapy. Brachytherapy. 2011;10(3):232‐241. doi:10.1016/j.brachy.2010.08.008 20932810

[8] Mikell JK , Klopp AH , Price M , Mourtada F . Commissioning of a grid‐based Boltzmann solver for cervical cancer brachytherapy treatment planning with shielded colpostats. Brachytherapy. 2013;12(6):645‐653. doi:10.1016/j.brachy.2013.04.007 23891341

[9] Boman EL , Satherley TWS , Schleich N , Paterson DB , Greig L , Louwe RJW . The validity of Acuros BV and TG‐43 for high‐dose‐rate brachytherapy superficial mold treatments. Brachytherapy. 2017;16(6):1280‐1288. doi:10.1016/j.brachy.2017.08.010 28967561

[10] Libby B , Ter‐Antonyan R , Schneider BF . Comparison of TG43 to Acuros dose calculations for high‐dose‐rate gynecological brachytherapy. Brachytherapy. 2011;10:S66. doi:10.1016/j.brachy.2011.02.122

[11] Iftimia I , Halvorsen PH . Commissioning of the Acuros BV GBBS Algorithm. Brachytherapy. 2015;14:S88. doi:10.1016/j.brachy.2015.02.349

[12] Peltola E . Numerical and Experimental Evaluation of Acuros BV Algorithm for Brachytherapy, Tampere University, 2022.

[13] Bellezzo M , Baeza JA , Voncken R , Reniers B , Verhaegen F , Fonseca GP . Mechanical evaluation of the Bravos afterloader system for HDR brachytherapy. Brachytherapy. 2019;18(6):852‐862. doi:10.1016/j.brachy.2019.06.005 31327634

[14] IROC Houston . User Guide for Varian Medical Systems BrachyVision Algorithm Testing. 2015.

[15] Ma Y , Vijande J , Ballester F , et al. A generic TG‐186 shielded applicator for commissioning model‐based dose calculation algorithms for high‐dose‐rate Ir‐192 brachytherapy. Med Phys. 2017;44(11):5961‐5976. doi:10.1002/mp.12459 28722180

[16] University of Wisconsin Accredited Dosimetry Calibration Laboratory . Description of Reported UWADCL Well‐Type Ionization Chamber Calibration Coefficients. University of Wisconsin; 2019.

[17] Ballester F , Puchades V , Lluch JL , et al. Technical note: Monte‐Carlo dosimetry of the HDR 12i and Plus sources. Med Phys. 2001;28(12):2586‐2591. doi:10.1118/1.1420398 11797964

[18] Taylor REP , Rogers DWO . EGSnrc Monte Carlo calculated dosimetry parameters for ^192^Ir and ^169^Yb brachytherapy sources. Med Phys. 2008;35(11):4933‐4944. doi:10.1118/1.2987676 19070227

[19] Perez‐Calatayud J , Ballester F , Das RK , et al. Dose calculation for photon‐emitting brachytherapy sources with average energy higher than 50 keV: Report of the AAPM and ESTRO. Med Phys. 2012;39(5):2904‐2929. doi:10.1118/1.3703892 22559663

[20] International Commission on Radiation Units and Measurements. J ICRU. 2013;13(1‐2):NP.2‐NP. doi:10.1093/jicru/ndw028 27335497

[21] Varian . *BrachyVision Algorithms Reference Guide*. Varian Medical Systems, Inc. 2021. www.MyVarian.com

[22] Mikell JK , Mourtada F . Dosimetric impact of an ^192^Ir brachytherapy source cable length modeled using a grid‐based Boltzmann transport equation solver. Med Phys. 2010;37(9):4733‐4743. doi:10.1118/1.3478278 20964191

[23] Papagiannis P , Pantelis E , Karaiskos P . Current state of the art brachytherapy treatment planning dosimetry algorithms. Br J Radiol. 2014;87(1041):20140163. doi:10.1259/bjr.20140163 25027247 PMC4453149

[24] Tien CJ , Chen ZJ . Deployment and performance of model‐based dose calculation algorithm in Ir‐192 shielded cylinder brachytherapy. Brachytherapy. 2019;18(6):883‐889. doi:10.1016/j.brachy.2019.07.006 31444132

[25] Chetty IJ , Curran B , Cygler JE , et al. Report of the AAPM Task Group No. 105: Issues associated with clinical implementation of Monte Carlo‐based photon and electron external beam treatment planning. Med Phys. 2007;34(12):4818‐4853. doi:10.1118/1.2795842 18196810

